# Emotions and the Right Hemisphere: Editorial

**DOI:** 10.3390/brainsci11121579

**Published:** 2021-11-29

**Authors:** Guido Gainotti

**Affiliations:** Institute of Neurology, Catholic University, Largo Agostino Gemelli 8, 00168 Rome, Italy; guido.gainotti@unicatt.it; Tel.: +39-06-3550-1945; Fax: +39-06-3550-1909

The hypothesis assuming that the right hemisphere may play a critical role in emotional processing was raised by clinical data which showed that patients with right brain lesions often show abnormal patterns of emotional behavior [1]. This hypothesis, however, quickly expanded to other fields of the affective neurosciences, giving rise to other models of emotional lateralization, such as the valence hypothesis [2] or the emotion-type hypothesis [3], and to attempts to identify right hemisphere structures that could play a critical role in the computation, communication, modulation, and experience of emotions [4]. Furthermore, other lines of research have tried to connect emotions to other brain functions in which the right hemisphere could play a leading role or to identify phylogenetic antecedents of the human right hemisphere dominance for emotions.

This Special Issue of *Brain Sciences* contains a well-matched combination of all these lines of research in the study of hemispheric emotional asymmetries. A first group of papers concern experimental and clinical investigations dealing with relevant aspects of the emotional communication or of the emotional behavior of patients with unilateral brain lesions, whereas a second group of articles consists of theoretical papers, which try to clarify controversial aspects of the emotional lateralization.

The first group of papers includes a technologically advanced study of emotional face processing through eye-tracking recording and a neuropsychological investigation of anhedonia (loss of pleasure) in semantic dementia patients with prevalent degeneration of the right or left anterior temporal lobes. 

In the first study, Hutchings et al. [5] have demonstrated more fixations on the eyes of emotional faces, in patients with right-sided (but not left left-sided) atrophy. This pattern was evident across all three emotional conditions (fear, happy, neutral) and was associated with degradation of the right superior temporal sulcus, which is important for processing changeable facial cues. 

In the second investigation, Shaw et al. [6] have shown that a prevalent atrophy of right hemisphere structures negatively impacts on the capacity to experience pleasure in semantic dementia patients. Regression and mediation analyses demonstrated a unique role for the right anterior temporal lobe (ATL) in the origin of anhedonia, in agreement with a number of studies implicating degeneration of the right ATL in the genesis of socioemotional disturbances in semantic dementia patients.

The third anatomo-clinical paper is the study of Durfee et al. [7], dedicated to an explicit training protocol targeting affective prosody recognition in adults with acute right hemisphere stroke and receptive aprosodia. This investigation has shown that several ventral stream regions and pathways of the right hemisphere can influence training effectiveness. 

Kliemann et al.’s paper is also dedicated to the brain networks which could compensate emotional disorders, resulting from right hemisphere damage [8]. It also deals with the compensatory reorganization of the social brain in individuals whose right cerebral hemisphere had been (to a large extent) surgically removed to treat epilepsy. Results of this study suggest that social brain networks typically distributed across midline and lateral brain regions can be reorganized to a substantial degree after right hemispherectomy.

If we pass from the anatomo-clinical to the theoretical papers, we see that many relevant problems are taken into account in this Special Issue, such as: (a) the relations between the right hemisphere dominance for emotions and other asymmetrical brain functions; (b) the lateralization of emotion regulation strategies; (c) the differences between basic and social emotions; and (d) the integration between human and animal studies on emotional lateralization.

Three of these papers try to identify the links that could exist between the right hemisphere dominance for emotions and other kinds of brain functions. Thus, in a series of hemianopic patients, Ladavas and Bertini [9] have shown that fearful faces are unconsciously processed via a subcortical colliculo–pulvinar–amygdala pathway, which facilitates responses towards visual stimuli in the intact visual field. This fear-specific implicit visual processing pathway has, however, been observed only after lesions to the left visual cortices, while no effect was found in patients with damage to the right hemisphere. This demonstrates the critical role of the right hemisphere in unconscious processing of emotionally salient visual stimuli, and forces us to consider the right amygdala as an integral component of a constant and continuous vigilance system, lying at the interface between emotional and attention-orienting mechanisms. 

Very similar positions are defended by Hartikainen [10], who reviews data showing that arousing emotional stimuli have prioritized access to right lateralized attention networks and that the right hemisphere ventral attention system is involved in shifting attention to salient stimuli. For this reason, in the right hemisphere, unattended emotional stimuli tend to be favored in attentional resource competition at the expense of task-relevant stimuli leading to interference in task performance.

On the other hand, Kheirkhah et al. [11] have taken into account the role of arousal in emotional processing, presenting healthy participants with high-arousing emotional (pleasant and unpleasant) and neutral pictures, and measuring their brain responses with magnetoencephalography, which provides higher spatial resolution than electroencephalography and higher temporal resolution than other neuroimaging technologies. These authors showed that the largest differences in response to high-arousing emotional versus neutral stimuli occurred in the right temporo-parietal region for most participants, regardless of whether the high-arousing stimuli were positive or negative.

It must also be noted that data reviewed by Ladavas and Bertini [9] and by Hartikainen [10], which stress the associations within the right hemisphere between emotional and attention-orienting mechanisms, are consistent with models (e.g., [12]) which assume that intrinsic links must exist between orienting and emotional mechanisms, because the capture of attention by possible sources of threat is usually the first step of the sequence of events that leads to the generation of the corresponding emotion.

Data relevant to the issue of the interactions between emotion generation and emotion regulation strategies have been carefully reviewed by Turnbull and Salas [13]. These authors make reference to the Gross’ [14] Process Model of Emotion Regulation, which proposes that human beings manage feelings (in a range of ways, from voluntarily to automatic) by using a wide range of regulatory strategies, which depend on diverse neuropsychological functions. After having systematically explored hemispheric asymmetries in the regulation of effect, Turnbull and Salas [13] maintain that, since emotion regulation strategies are based on complex cognitive processes, the question cannot concern the lateralization of the whole regulation process, but the lateralization of the specific cognitive tools we use to manage our feelings, in a range of different ways. 

Ross [15] advances in his paper the hypothesis that emotions may be classified into primary and social types and that hemispheric lateralization may be better explained by the emotion-type hypothesis, which posits that only primary emotions may be modulated by the right hemisphere, whereas social emotions might be modulated by the left hemisphere and, in particular, by the left prefrontal cortex. This neocortical region matures, indeed, later than other cortical areas and is involved in complex, higher-order, social–emotional, and executive functions.

Finally, Gainotti [16] takes into account the problem of the difficult integration between human and animal studies on emotional lateralization. Data from his review suggest that, in these studies, there are both elements of continuity and factors of discontinuity between the emotional asymmetries observed in animals and humans. The right lateralization of sympathetic functions (involved in brain and bodily activities necessary in emergency situations typical of primary emotions) could be consistent across many animal species, whereas asymmetries in emotional communication and in emotional experience, similar to those observed in humans, could be present only in higher primates.

Taken together, papers gathered in this Special Issue of *Brain Sciences* dealing with ‘Emotions and the Right Hemisphere’ should, therefore, be of great interest for neuroscientists interested in clinical, neuroanatomical, and cognitive aspects of effective and social neurosciences.
